# Clinical effect and safety of continuous renal replacement therapy in the treatment of neonatal sepsis-related acute kidney injury

**DOI:** 10.1186/s12882-020-01945-z

**Published:** 2020-07-18

**Authors:** Cheng Cai, Gang Qiu, Wenchao Hong, Yunlin Shen, Xiaohui Gong

**Affiliations:** Department of Neonatology, Shanghai Children’s Hospital, Shanghai Jiao Tong University, No.355, Luding Road, Shanghai, 200062 China

**Keywords:** Continuous renal replacement therapy, Acute kidney injury, Neonates, Efficacy, Safety

## Abstract

**Background:**

Sepsis is the leading cause of acute kidney injury (AKI) in the neonatal intensive care unit (NICU). The aim of the study is to explore the efficacy and security of continuous renal replacement therapy (CRRT) in the treatment of neonatal sepsis-related AKI.

**Method:**

Totally12 sepsis-related AKI neonates treated with CRRT were hospitalized in the NICU of Shanghai Children’s Hospital between November 2012 and November 2019, and the clinical data of these 12 cases were retrospectively analyzed. Renal function, acid-base balance, electrolytes, blood pressure and hemodynamics indexes were recorded before CRRT initiation, 12/24/48 h after CRRT initiation and at the end of CRRT respectively. The efficacy of CRRT was evaluated and the clinical outcome was observed in these 12 sepsis-related AKI neonates. Repeated measurement analysis of variance was used for statistical analysis of the data.

**Result:**

(1) Continuous veno-venous hemodialysis filtration (CVVHDF) was used in 12 cases of sepsis-related AKI neonates. There were 6 cases with oliguria, 3 cases with fluid overload (FO), 3 cases with septic shock. The duration of CRRT was 49 ~ 110 h, average (76.2 ± 23.5) h. (2) The blood pressure (BP) of 12 sepsis -related AKI neonates could reach the normal level (40–60 mmHg) 12 h after CRRT initiation, and the normal BP level could be maintained during the CRRT treatment. After 12 h CRRT, the blood pH value increased to the normal range (7.35 ~ 7.45). After 12 h CRRT treatment, the oxygenation index of 12sepsis-related AKI neonates could reach 200 mmHg. After 24 h CRRT treatment, it could rise to more than 300 mmHg. Serum potassium, serum urea nitrogen and serum creatinine levels decreased significantly 12 h after CRRT initiation, and reached the normal range 24 h after CRRT initiation. The urine volume significantly increased 24 h after CRRT initiation. (3) Venous catheterization was performed successfully in all sepsis-related AKI neonates. We observed 2 cases of thrombocytopenia, 1 case of obstruction and 1 case of hypotension in the course of CRRT. There were no complications such as hypothermia, hemorrhage, thrombosis and infection.11 neonates were cured and discharged. One neonate was treated with CRRT and passed through the oliguria stage of AKI, but died after the parents gave up the treatment.

**Conclusions:**

It is safe and effective to treat neonatal sepsis-related AKI with CRRT, which should be an effective measure for the treatment of sepsis-related AKI neonates.

## Background

Neonatal sepsis is a life-threatening organ dysfunction caused by the maladjustment of the newborn’s response to infection, which is one of the main causes of neonatal death in NICUs [1, 2]. Sepsis-related AKI refers to the impairment of renal function of neonates with sepsis in a short time [3]. Sepsis-related AKI is one of the main causes for the high mortality in sepsis. Increased release of various inflammatory cytokines and local inflammation in kidney play important roles in the development of AKI. It has been reported that if sepsis-related AKI occurs in children (sepsis accounts for 50% of AKI causes), the mortality rate is as high as 70%, which is far higher than that of non sepsis-related AKI(51.8%). At this time, AKI is considered as an independent risk factor and affects the prognosis and mortality of children with sepsis [4]. At present, there is no report on the mortality of neonatal sepsis-related AKI, and no effective prevention and treatment for neonatal sepsis-related AKI. In recent years, the scope of continuous renal replacement therapy (CRRT) has been applied from children’s AKI to neonatal AKI [5]. However, there is still a lack of large data from multicenter on CRRT in the treatment of neonatal sepsis-related AKI. The purpose of this study is to investigate the efficacy and safety of CRRT in the treatment of neonatal sepsis-related AKI.

## Methods

### Study design and patient population

This clinical research was conducted in the NICU of Shanghai Children’s Hospital between November 2012 and November 2019. The Institutional Review Board (IRB) at Shanghai Children’s Hospital approved the study protocol (approval No. 2012C001-F01). Neonates who exhibited any of the comorbidities associated with AKI and sepsis were enrolled in this investigation. The following risk factors were considered to be associated with AKI and neonatal sepsis: (1) The age of admission was less than 28 days, (2) The weight of admission was more than 2.0 kg, (3) Sepsis was confirmed, (4) Sepsis-related AKI. All parents of the neonates provided written informed consent to join the study. Overall, 42 AKI neonates were included initially. Non sepsis neonates (*n* = 30) were excluded.

In all 12 sepsis-related AKI neonates, 7 males and 5 females, the gestational age (GA) of was 33^+ 4^ ~ 40^+1^weeks, and the age of admission was 2 ~ 28 days. The birth weight was 2250 ~ 4000 g, with an average of (3050 ± 450) g. Five cases (including 3 cases of neonatal septic shock) were treated by endotracheal intubation and ventilator -assisted ventilation, 4 cases were treated by nasal continuous positive airway pressure (NCPAP), and the other 3 cases were not treated by oxygen inhalation. (Additional file 1).

### Data collection

Retrospective analysis collected database was used to examine the following variables during NICU admission: demographic characteristics, primary diagnosis, renal function, acid-base balance, electrolytes, blood pressure and hemodynamics indexes were recorded before CRRT initiation, 12/24/48 h after CRRT initiation and at the end of CRRT respectively. The efficacy of CRRT was evaluated and the clinical outcome was observed in these 12 sepsis-related AKI neonates. The primary study endpoint was clinical outcome in order to determine the efficacy and safety of CRRT in the treatment of neonatal sepsis-related AKI.

### Diagnostic criteria

Neonatal sepsis: Reference literature [1, 6]:

1. Clinical diagnosis: there are clinical abnormal manifestations, and all the following conditions meet at the same time: ①more than 2 nonspecific blood examinations are positive; ②cerebrospinal fluid examination was purulent meningitis change; ③pathogenic bacteria DNA is detected in blood.
2. Definite diagnosis: clinical manifestations, any positive result of microorganism cultures.

Neonatal AKI [7]: At present, according to the standards of AKI clinical practice guidelines issued by the Global Committee for Improving the Prognosis of Kidney Disease (KDIGO) in 2013, the diagnosis of neonatal AKI mainly depends on the change of serum creatinine (CR) and urine volume. (Table 1).

**Table 1:** Neonatal KDIGO (Kidney Diseases: Improving Global Outcomes) acute kidney injury definition

Stage	Serum creatinine (SCr)	Urine output over 24 h
0	No change in serum creatinine or rise < 0.3 mg/dL	> 1 mL/kg/h
1 2 3	SCr rise ≥0.3 mg/dL within 48 h or SCr rise ≥1.5 to 1.9 × reference SCr ^a^ within 7 days SCr rise ≥2 to 2.9 × reference SCr ^a^ SCr rise ≥3 × reference SCr ^a^ or SCr ≥2.5 mg/dL ^a^ or Receipt of dialysis	> 0.5 and ≤ 1 mL/kg/h > 0.3 and ≤ 0.5 mL/kg/h ≤0.3 mL/kg/h

^a^ Reference SCr is the lowest prior SCr measurement

Neonatal sepsis-related AKI [8]: meet both the diagnostic criteria for sepsis and AKI and exclude other causes for AKI.

Fluid overload (FO): gain weight10% or more compared with admission weight.

### CRRT treatment

Indications of CRRT: Neonatal sepsis-related AKI stage 0 with FO, or Cr, electrolyte disorder or internal environment change was not obvious after regular treatment of AKI stage 1 and above, and coagulation function was normal or nearly normal [9].

Contraindications of CRRT: (1) Gestational age < 34 weeks, or body weight < 2.0 kg, (2) Non sepsis, (3) Non AKI, (4) Hypotension: as for hypotension, CRRT should be initiated after the blood pressure rises, (5) Bleeding tendency: CRRT could be performed after the coagulation function was partially corrected, or anticoagulant application could be reduced according to the coagulation function of children, (6) Intracranial hemorrhage: especially grade 3 and grade 4 intracranial hemorrhage, (7) Bleeding of important organs in vivo: CRRT could only be performed after hemostasis, and local anticoagulation in vitro with citric acid was used for treatment.

Method of CRRT: (1) Operating methods: The equipment was Plasauto iQ21. CVVHDF mode was selected according to the molecular weight of solute removal. CRRT instrument consists of two parts: external blood circulation and filter. The specific operation processes were as follows: heparin saline was pre-filled with external blood circulation and filter, and then 68 ml red cell suspension was used to pre-fill the external blood circulation and filter. CRRT has a blood circulation volume of 38 ml (arterial line + venous line). It was purchased from Japan Laifuen Co., Ltd. The filter capacity is 30 ml. For CRRT, 5Fr, single-tube and double-chamber central venous catheters were used. The arterial hole was at the telecentric end, and the venous hole was at the proximal end, which were 2 to 3 mm apart. The blood recirculation was less than 10%. Common puncture sites are femoral vein, internal jugular vein, and umbilical vein, which can be used in neonates of age under 7 days. Connect the blood purifier pipeline for bypass. (2) CRRT parameters: initial flow rate of blood pump was 3 ml /(kg. min), and then was increased to 5 ml /(kg min) according to blood pressure (BP). The flow rate of replacement fluid was 20–30 ml /(kg. h), and for dialysate, the flow rate was 15–25 ml /(min.m^2^). Dehydration speed = filter pump - dialysis pump - rehydration pump, uninterrupted flow. (3) Dialysis fluid and replacement fluid: Baxter dialysate was used in dialysate (Baxter Company). The replacement solution was prepared by NICU nurses in our unit. The Ports scheme was adopted to improve the formula, adding 5% glucose solution 100 ml, 10% calcium chloride solution 7.5 ml, 50% magnesium sulfate solution 1.6 ml and 5% sodium bicarbonate solution 200 ml in Ringer’s solution 3000 ml. The ionic concentration of the formula contained 130.0 mmol/L sodium ion, 4.0 mmol/L potassium ion, 28.0 mmol/L bicarbonate ion, 1.5 mmol/L calcium ion, 3.2 mmol/L magnesium ion and 109.0 mmol/L chloride ion. In this formula, the concentration of glucose is 0.2 g/L. Ion concentration was adjusted according to electrolyte monitoring. (Additional file 1).

Maintenance of CRRT: (1) anticoagulation: the filter and pipeline were pre-filled with heparin solution. Heparin anticoagulation was used to maintain prothrombin time (PT) at 25–40 s and activated partial thromboplastin time (APTT) at 80–120 s. The dosage of heparin was generally 5-40 U/(kg.h). (2) Replacement of filter membranes: if blockage occurs during treatment, the filter membranes should be replaced in time. Indications for terminating CRRT: If urine volume of neonates with sepsis-related AKI was more than 2 ml/(kg·h), or FO was obviously improved, and no electrolyte and acid-base balance disorders occurred, CRRT was terminated. (Additional file 1).

### Observation item

1. Clinical manifestations and pathogens of neonatal sepsis.
2. The changes of serum potassium, sodium, urea nitrogen, creatinine and urine volume were observed before the initiation of CRRT, 12/24/48 h after the initiation of CRRT and at the end of CRRT.
3. Clinical outcomes of 12 sepsis-related AKI neonates were recorded.
4. The occurrence of CRRT - related complications, such as pipeline blockage, hemorrhage, hypothermia, thrombosis, infection and thrombocytopenia were analyzed statistically.

### Evaluation of curative effect

We evaluated the effectiveness by analyzing the changes of urine volume, renal function, blood electrolyte and acid-base balance before and after CRRT. If these indicators were obviously improved, it turned out to be effective. If there was no improvement, it would be ineffective.

### Evaluation of safety

We analyzed the difficulty of intravenous catheterization in sepsis-related AKI neonates and the occurrence of CRRT -related complications to evaluate the safety of CRRT.

### Statistical analysis

SPSS 22.0 statistical software was used to analyze the data. The data of measurement data was conformed to the normal distribution through the normal test, so it was expressed bymean ± standard deviation (x ± s). Repeated measurement analysis of variance was used to compare the time points between groups or within the same group. *P* < 0.05 was statistically significant.

## Results

### Catheters site and size

All 12 cases of CRRT were treated with 5Fr catheter. Three cases were placed in the internal jugular vein and 9 cases in the femoral vein. CVVHDF was used in 12 cases of sepsis-related AKI. The mean time of CRRT treatment was (76.2 + 23.5) h.

### Clinical manifestations and pathogens of neonatal sepsis

Neonatal sepsis might show non-specifc signs and symptoms or focal signs of infection, including temperature instability, poor perfusion with pallor and mottled skin, metabolic acidosis, tachycardia or bradycardia, apnoea, respiratory distress, feeding intolerance, abdominal distention, jaundice, and so on.

The results of blood culture of 12 neonates with sepsis were positive in 7 cases and negative in 5 cases. The positive pathogens in 7 cases were 3 cases of *Streptococcus agalactiae* (GBS), 2 cases of *Escherichia coli*, 1 case of Klebsiella pneumonia, and 1 case of *Enterococcus faecium*.

### Changes of renal function and electrolyte indicators before and after CRRT treatment

**Fig. 1:**
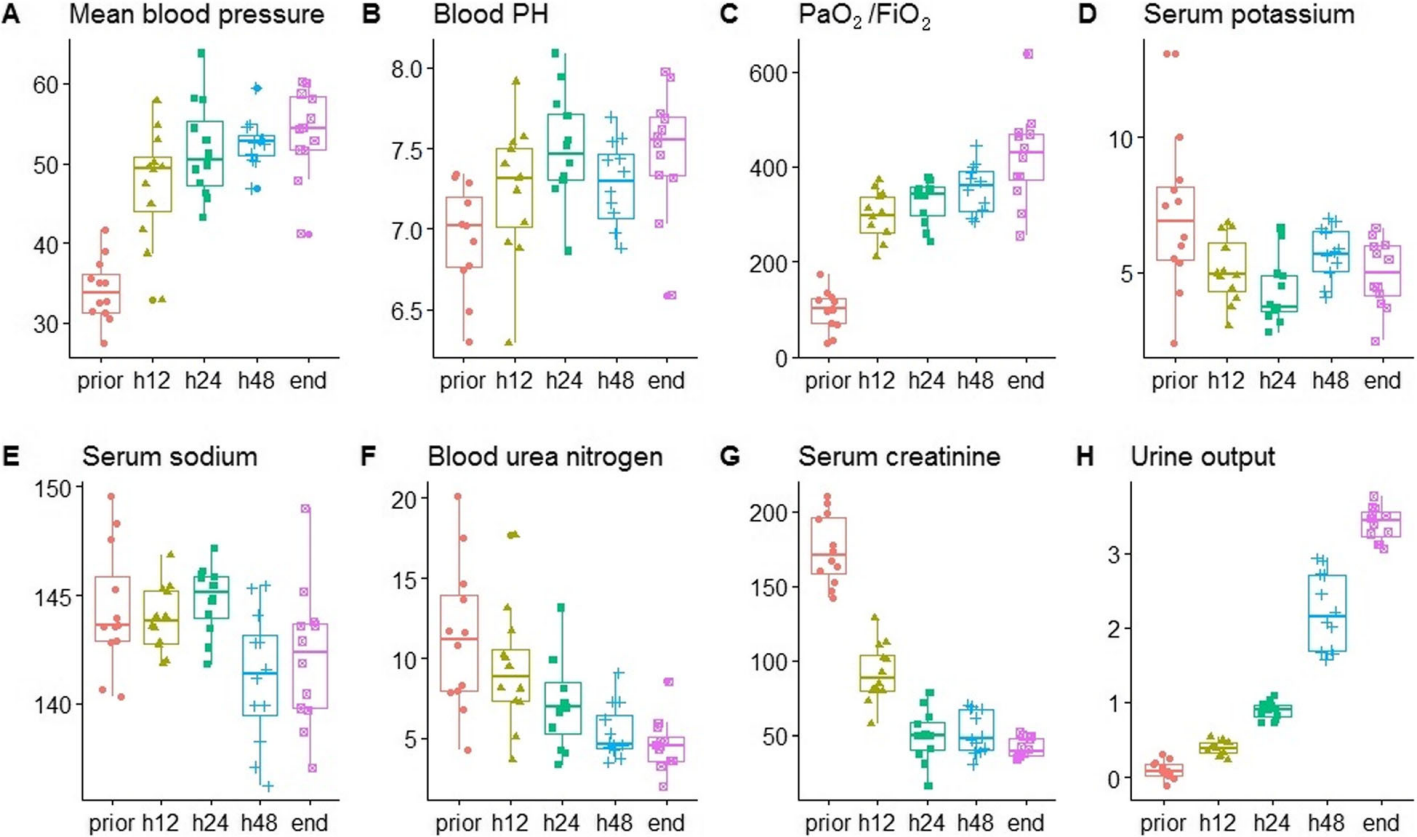
Changes of indexes related to cardiopulmonary function and renal function and electrolyte of 12 sepsis related AKI neonates during CRRT therapy. **a** Mean blood pressure, **b** blood PH, **c** PaO_2_/FiO_2_, **d** Serum potassium, **e** Serum. Sodium, **f** Blood urea nitrogen, **g** Serum creatinine, **h** Urine output. In addition to serum sodium, indexes related to cardiopulmonary function and renal function and electrolyte showed statistically significant changes at different treatment time points of CRRT

1. Blood pressure (BP): After 12 h of CRRT, BP of 12sepsis-related AKI neonates could reach the normal level (40–60 mmHg), and the normal BP level could bemaintained during CRRT.(Fig. 1, Table 2)
2. Blood pH value: After 12 h of CRRT combined with sodium bicarbonate injection in 12 cases of sepsis-related AKI, blood pH value increased to the normal range (7.35 ~ 7.45). The dosage of sodium bicarbonate injection was diluted according to the absolute value of residual alkali and the ratio of 1:2.5 or above.(Fig. 1, Table 2)
3. PaO_2_/FiO_2_: refers to the ratio of arterial partial pressure of oxygen (PaO_2_) to inhaled oxygen concentration (FiO_2_). After 12 h of CRRT, the oxygenation index of 12sepsis-related AKI neonates could reach 200 mmHg. After 24 h of CRRT, it could rise to more than 300 mmHg.(Fig. 1, Table 2)
4. Indicators of renal function and electrolytes: Serum potassium, serum urea nitrogen and serum creatinine levels decreased significantly after 12 h of CRRT, and reached the normal range after 24 h of CRRT. After 24 h of CRRT, the urine volume significantly increased.(Fig. 1, Table 3)
5. Fluid balance: The urine volume significantly increased 24 h after CRRT initiation. 3 cases of neonates with FO significantly improved after CRRT treatment. (Fig. 1, Table 3)

**Table 2:** Changes of indexes related to cardiopulmonary function of 12 sepsis related AKI neonates before and after CRRT therapy (x ± s)

Time points	Mean blood pressure (mmHg)	Blood PH	PaO_2_/FiO_2_(mmHg)
Prior treatment	34.6 ± 6.4	7.00 ± 0.35	94.2 ± 45.8
12 h of treatment	47.6 ± 5.3^a^	7.27 ± 0.34^a^	301.6 ± 46.7^a^
24 h of treatment	51.4 ± 6.2^a^	7.41 ± 0.32^a^	323.5 ± 55.2^a^
48 h of treatment	53.6 ± 5.1^a^	7.39 ± 0.26^a^	354.3 ± 62.6^a^
End of treatment	55.2 ± 4.8^a^	7.43 ± 0.35^a^	411.2 ± 61.4^a^
*H* Value	33.195	3.237	54.776
*P* Value	0.000	0.025	0.000

Note: ^a^ compared with before treatment, *P* < 0.05; 1 mmHg = 0.133 kPa

**Table 3:** Changes of renal function and electrolyte indexes of 12 sepsis related AKI neonates before and after CRRT therapy (x ± s)

Time points	Serum potassium (mmol/L)	Serum sodium (mmol/L)	Blood urea nitrogen (mmol/L)	Serum creatinine (μmol/L)	Urine output [ml/(kg•h)]
Prior treatment	6.4 ± 3.1	141.7 ± 3.6	14.6 ± 5.4	174.6 ± 23.5	0.1 ± 0.1
12 h of treatment	5.1 ± 1.5^a^	143.5 ± 2.5	9.6 ± 3.5^a^	96.8 ± 17.3^a^	0.4 ± 0.1
24 h of treatment	4.5 ± 1.6^a^	144.3 ± 1.7	7.6 ± 2.4^a^	61.4 ± 18.7^a^	0.9 ± 0.1^a^
48 h of treatment	5.0 ± 1.4^a^	141.0 ± 2.5	5.5 ± 1.6^a^	45.3 ± 10.3^a^	2.0 ± 0.5^a^
End of treatment	4.7 ± 1.3^a^	142.4 ± 2.8	5.0 ± 1.4^a^	41.2 ± 9.4^a^	3.3 ± 0.2^a^
*H* Value	0.967	2.335	41.204	140.983	415.922
*P* Value	0.003	0.070	0.000	0.000	0.000

Note:Compared to before CRRT treatment, ^a^*P* < 0.05

### Evaluation of the effectiveness, feasibility and safety of CRRT

1. Effectiveness: 12 cases of sepsis-related AKI neonates were treated effectively. It showed that the renal function, urine volume, blood electrolyte, acid-base balance and hemodynamics were significantly improved after treatment.
2. Feasibility and safety: Venous catheterization was performed successfully in 12 cases of sepsis-related AKI neonates. Thrombocytopenia occurred in 3 cases, besides, there was 1 case of obstruction and 1 case of hypotension in the course of CRRT. There were no complications, such as hypothermia, hemorrhage, thrombosis and infection.

### Clinical outcome

Eleven cases of sepsis-related AKI neonates were cured and discharged. One case was treated with CRRT and passed through the oliguria stage of AKI, but the neonate died after the parents giving up the treatment.

## Discussion

Acute kidney injury (AKI) is associated with high mortality in critically ill neonates, especially neonates with sepsis and septic shock. Many researches show that more than 50% of AKI in ICU is related to sepsis [10]. At present, many studies believe that AKI may occur even when there is no decrease or even increase in renal blood flow perfusion in sepsis-related AKI [11]. Inflammatory factors storm and cell apoptosis may play an important role in the pathogenesis of sepsis-related AKI [12]. Therefore, it is more appropriate to classify sepsis-related AKI as renal AKI than as pre-renal AKI.

Recently, with the rapid development of CRRT technology, CRRT plays a key role in the treatment of neonatal AKI and even critically ill neonates, which can significantly improve the efficacy and prognosis of sepsis-related AKI neonates [13]. CRRT, also known as continuous blood purification (CBP), is commonly used to provide renal support for critically ill patients with AKI, particularly patients who are hemodynamically unstable [14]. The main principles of CRRT are dispersion, convection, adhesion and adsorption [15]. In 1985, Ronco et al. [16] reported the successful application of continuous arteriovenous hemofiltration (CAVH) in the treatment of neonatal AKI. From then on, a new starting point of CRRT in the treatment of neonatal AKI was opened.

In this study, there were 6 cases of oliguria: 3 cases of fluid overload (FO) and 3 cases of septic shock. FO is associated with adverse outcomes in critical illness and often represents a primary indication for CRRT [17, 18]. Blood pressure (BP) could reach the normal level after 12 h of CRRT, and it could be maintained in the normal range during the treatment. This shows that CRRT can maintain the hemodynamic stability of neonates of sepsis-related AKI at an earlier stage. After 12 h of CRRT, the blood pH value increased to the normal range of 7.35–7.45. After 12 h of CRRT, the blood potassium, blood urea nitrogen and blood creatinine decreased significantly, and recovered to the normal level after 24 h of treatment. After 24 h of CRRT, the urine volume of neonates increased significantly. These results show that CRRT can effectively remove a large number of inflammatory mediators, dialyse out a large amount of excess water, significantly improve renal function, and maintain the stability of the internal environment. This study shows that PaO_2_/FiO_2_ of the children can reach 200 mmHg at 12 h and more than 300 mmHg at 24 h. The results showed that CRRT could improve tissue oxygenation and metabolism of sepsis-related AKI neonates by eliminating pulmonary interstitial edema, improving microcirculation and increasing the oxygen uptake capacity of parenchymal cells.

Zhang et al. [19] research has shown that CRRT could not only maintain fluid balance in vivo, excrete metabolites, but also clear inflammatory factors and promote renal recovery. The mechanism of CRRT in the treatment of sepsis-related AKI is as follows [20–22], CRRT can regulate renal metabolic adaptation and restoring renal function. Therefore, CRRT can not only deal with water balance and metabolites, but also can improve tissue oxygenation and metabolism of sepsis-related AKI neonates [23]. In this study, we found that after 24 h of CRRT, the levels of blood urea nitrogen and creatinine decreased significantly. In 2016, when the demand for metabolism and fluid management exceeds the capacity of kidney, which is called fluid overload, it should be treated as early as possible [24]. This means that when the metabolism and fluid management of sepsis-related AKI are beyond the renal capacity, (even without renal damage), CRRT should be initiated in time.

The most important index of the feasibility and safety of CRRT is the difficulty of catheterization and complications. There are some complications in CRRT in the treatment of sepsis-related AKI neonates, such as difficulty or failure of catheterization, thrombocytopenia, blockage of blood vessels, hypotension, hypothermia, thrombosis, hemorrhage and blood flow infection [25]. In this study, venous catheterization was performed successfully in 12 cases sepsis-related AKI neonates. There were 2 cases of thrombocytopenia, 1 case of obstruction and 1 case of hypotension in the course of CRRT. There were no complications such as hypothermia, hemorrhage, thrombosis and infection. According to the analysis, the causes of thrombocytopenia might include sepsis or septic shock, anticoagulant, and disseminated intravascular coagulation. The causes of hypotension might be volume-related factors, such as too fast dehydration rate. The causes of pipeline blockage might be slow blood flow rate, poor anticoagulation control, etc. Skillful puncture technique, timely close monitoring and strict aseptic operation are the key to reduce or prevent the complications of CRRT [26]. During CRRT treatment in our department, blood coagulation function, blood gas analysis and micro blood glucose were strictly monitored every 2–4 h. Liver function, renal function and blood electrolyte were detected every 6–12 h. In order to avoid or reduce the complications of CRRT, it is necessary to monitor the vital signs of neonates and detect the indexes such as the balance of the volume of blood, the function of blood coagulation, hemodynamics, blood electrolyte and blood glucose.

In this study, 11 cases of sepsis-related AKI neonates were cured and discharged, 1 case died after the parents giving up the treatment. Symons et al. [27] conducted a retrospective study on the CRRT data of 85 children with body weight of less than 10 kg in five hospitals in the United States. The study included 13 cases of multiple organ dysfunction (MODS), 12 cases of sepsis, 16 cases of children whose weight was less than 3.0 kg, and the minimum weight was 1.5 kg. The results of this study showed that the effect and prognosis of CRRT were similar among children of 3.0–10.0 kg or in elder children. It can be seen that CRRT may significantly improve the clinical outcome and prognosis of AKI in low birth weight infants [28].

## Conclusions

In summary, it is safe and effective to treat neonatal sepsis-related AKI with CRRT, which should be an effective measure for the treatment of sepsis-related AKI neonates. However, this is a single-center retrospective clinical study with few samples. In the future, we should carry out a multicenter clinical study of CRRT with more samples in the treatment of sepsis-related AKI neonates, and further explore the individualized accurate treatment of CRRT in the treatment of sepsis-related AKI neonates.

## Supplementary information

**Additional file 1.**

## Data Availability

The datasets used and/or analysed during the current study are available from the corresponding author on reasonable request.

## Abbreviations

AKI: Acute kidney injury
APTT: Activated partial thromboplastin time
BP: Blood pressure
CAVH: Continuous arteriovenous hemofiltration
Cr: Creatinine
CRRT: Continuous renal replacement therapy
CVVHDF: Continuous veno-venous hemodialysis filtration
FiO_2_: Inhaled oxygen concentration
FO: Fluid overload
GA: Gestational age
IRB: Institutional review board
MODS: Multiple organ dysfunction
NCPAP: Nasal continuous positive airway pressure
NICU: Neonatal intensive care unit
PaO_2_: Arterial partial pressure of oxygen
PT: Prothrombin time
